# LncRNA GAS5 as miR-26a-5p Sponge Regulates the PTEN/PI3K/Akt Axis and Affects Extracellular Matrix Synthesis in Degenerative Nucleus Pulposus Cells *in vitro*

**DOI:** 10.3389/fneur.2021.653341

**Published:** 2021-08-03

**Authors:** Liang Tan, Yifang Xie, Ye Yuan, Kai Hu

**Affiliations:** Department of Spine Surgery, The Affiliated Zhuzhou Hospital of Xiangya Medical College, Central South University, Zhuzhou, China

**Keywords:** intervertebral disc degeneration, nucleus pulposus cells, lncRNA GAS5, miR-26a-5p, PTEN, extracellular matrix, PI3K/Akt pathway

## Abstract

The role of lncRNA growth arrest specific 5 (GAS5) in degenerative nucleus pulposus cell (NPC) apoptosis has been reported, but the mechanism of GAS5 in extracellular matrix (ECM) synthesis in intervertebral disc degeneration (IDD) remains unknown. We aimed to investigate the mechanism of GAS5 in ECM synthesis in degenerative NPCs. GAS5 expression was measured in degenerative NPCs (CP-H170) and normal NPCs (CP-H097). siRNA-mediated GAS5 knockdown was transfected to NPCs to detect cell viability and the expression of ECM-related genes (Collagen II, aggrecan, Collagen I, and MMP-3). Subcellular localization of GAS5 was analyzed. The downstream gene and pathway of GAS5 in degenerative NPCs were explored. As our results indicated, lncRNA GAS5 was upregulated in degenerative NPCs. Silencing GAS5 improved the viability of degenerative NPCs and increased ECM synthesis. GAS5 was mainly located in the cytoplasm of NPCs. LncRNA GAS5 sponged miR-26a-5p to regulate PTEN. Overexpression of miR-26a-5p promoted ECM synthesis in degenerative NPCs. Akt inhibitor LY294002 reversed the promotion of silencing GAS5 on ECM synthesis of degenerative NPCs. In conclusion, lncRNA GAS5 sponged miR-26a-5p to upregulate PTEN and inhibit the PI3K/Akt pathway, thus inhibiting ECM synthesis of degenerative NPCs.

## Introduction

Intervertebral discs (IVD) are composed of nucleus pulposus (NP), annulus fibrosus, and cartilaginous endplates (1). The degenerative changes of IVD are related to the injury of adjacent structures, resulting in functional impairment; more vulnerability to injury; and clinical symptoms such as spinal stenosis, axial back pain, myelopathy, or radiculopathy (2). Intervertebral disc degeneration (IDD) is the primary pathological mechanism which underlies low back pain, leading to a great burden on the global health care system (3–5). IDD is an age-dependent and chronic molecular degeneration process where NP cell (NPC) senesce and the balance of extracellular matrix (ECM) synthesis and catabolism are disrupted (6). Composed of collagens and proteoglycans, the ECM of the inner disc maintains IVD normal function (7). With the increase of IDD during the process of disc degeneration, the synthesis of Collagen I and proteoglycan and ECM degradation also increase (8–10). Some biological technologies have been developed to prevent early disc degeneration by promoting ECM repair and regeneration (11). Therefore, it is imperative to identify the effect of ECM synthesis for IDD management.

The long noncoding RNAs (lncRNAs) are RNAs with a length of more than 200 nucleotides, which have diverse biological functions, including regulating cellular phenotypes and gene expression (12). Numbers of lncRNAs are differentially expressed in human degenerative NPCs and implicated in several pathological processes during IDD, including ECM balance, inflammatory responses, and apoptosis (13). GAS has become the focus of research topics recently, especially in various types of cancer because it is associated with the regulation of proliferation and apoptosis. GAS5 promotes PTEN expression and inhibits the PI3K/AKT pathway by regulating miR-21 expression in ischemic brain injury (14). Additionally, GAS5 represses the proliferation of cardiac fibroblast in atrial fibrillation by inhibiting ALK5 (15). However, the role of GAS5 in IDD has been rarely reported. LncRNA GAS5 could enhance the apoptosis of primary NPCs derived from patients with IDD (16). Additionally, NR_002578, an lncRNA transcribed from GAS5 is strongly expressed in degenerative NPCs (17, 18). Furthermore, lncRNAs act as competing endogenous RNAs (ceRNAs) to sponge microRNAs (miRs) in cancers (19). For example, lncRNA GAS5 modulates the osteogenic differentiation and calcification of human vascular smooth muscle cells *via* the miR-26a-5p/PTEN axis (20). miRs play critical roles in cell differentiation, proliferation, and survival by targeting mRNAs, and emerging evidence supports the role of miRs in ECM degradation and IDD processes (21, 22). PTEN promotes IDD by regulating NPC behaviors (23), and miR-26a-5p was reported to target PTEN in various cells including cardiomyocytes, the human umbilical vein, and endothelial cells (24–26). However, investigations into the interactions between miRs and GAS5 in IDD are scarce. Therefore, we speculate that lncRNA GAS5 may monitor IDD progression by competing with a potential miR. Consequently, we performed a series of histological and molecular experiments to identify the underlying mechanism of GAS5 in IDD, with the purpose to provide some novel therapies for IDD management.

## Materials and Methods

### Cell Culture and Transfection

Degenerative NPCs and normal NPCs provided by Procell Life Science and Technology Co., Ltd. (Wuhan, Hubei, China) (CP-H170, CP-H097) were cultured in Dulbecco's modified Eagle's medium-F12 medium containing 20% fetal bovine serum at 37°C with 5% CO_2_. The cells at passage 3 were taken for subsequent experiments.

The interference and negative control (NC) of GAS5 [small interfering RNA (siRNA) si-GAS5, si-NC], miR-26a-5p mimic, miR-NC and miR-26a-5p inhibitor, and inhibitor-NC were synthesized by Gene Pharma (Shanghai, China). The synthesized vectors were transfected into degenerative NPCs using Lipofectamine 3000 (Thermo Fisher, Waltham, MA, USA). Cells were pre-treated with 25 μm (27) Akt inhibitor LY294002 (S1737-5mg; Beyotime Biotechnology Co., Ltd., Shanghai, China) for 6 h and transfected with si-GAS5/si-NC. The following experiments were conducted at 48 h post the transfection.

### Immunocytochemistry

Climbing sheets of degenerative NPCs and normal NPCs at passage 3 were fixed with 4% paraformaldehyde (Beyotime) for 30 min at room temperature, washed with PBS, and immersed in 20% H_2_O_2_ methanol solution for 30 min at room temperature. After blockade with 1:10 horse serum (Beyotime) for 20 min at room temperature, cells were incubated with the Col II primary antibody (1:500, ab34712, Abcam, Cambridge, MA, USA) at 4°C overnight. A secondary antibody IgG (1:2,000, ab205718, Abcam) was subsequently added and incubated for 1 h at room temperature, followed by incubation with SABC reagent (Fuzhou Maixin Biotech Co., Ltd., Fuzhou, China) for 20 min in a 37°C thermostat water bath. Following DAB (Zhongshan Golden Bridge, Beijing, China) color development, hematoxylin (Beyotime) was added for counterstaining and observed under a microscope (TS100, Nikon, Tokyo, Japan).

### Reverse Transcription Quantitative Polymerase Chain Reaction

Total RNA from cells was isolated using TRIzol (Invitrogen, Carlsbad, CA, USA), and reverse-transcribed into cDNA using a reverse transcription kit (Thermo Fisher). The qPCR was performed using SYBR Green PCR kit (Applied Biosystems, Foster City, CA, USA). The PCR primers are presented in Table 1. GAPDH or U6 served as loading control. Data were analyzed by using the 2^−ΔΔCt^ method.

**Table 1:** Primer sequences of RT-qPCR.

**Gene**	**Forward (5^**′**^-3^**′**^)**	**Reverse (5^**′**^-3^**′**^)**
*LncRNA GAS5*	CTTGCCTGGACCAGCTTAAT	CAAGCCGACTCTCCATACCT
*NADPH*	TTAGGTGGAGCTCAGGCAGT	CTTTGCAGGTAGGGCAGAAG
*miR-26a-5p*	ACACTCCAGCTGGGTTCAAG TAATCCAGGA	TGGTGTCGTGGAGTCG
*PTEN*	CCATAACCCACCACAG	CAGTCCGTCCTTTCC
*CollagenII*	TCCCCTACCCCGCACTTC	GGGGGCCAATGGGACCTGTC
*aggrecan*	TGAGCGGCAGCACTTTGAC	TGAGTACAGGAGGCTTGAG
*CollagenI*	CCTGGCAATATTGG TCCCGT	GATGTCCAGTGCGACCATCT
*MMP-3*	TTCCGCCTGTCTCAAG ATGATAT	AAAGGACAAAGCAGGATCA CAGTT
*U6*	CTCGCTTCGGCAGCACA	AACGCTTCACGAATTTGCGT
*GAPDH*	GGCACAGTCAAGGCTGA GAATG	ATGGTGGTGAAGACGCCAGTA

### Cell Counting Kit-8 Assay

Single cell suspension was plated into 96-well-plates (1 × 10^4^ cells/well, 100 μl/well). When culturing for 24, 48, and 72 h, each well was added with a 10 μl CCK-8 solution (Dojindo, Kumamoto, Japan) and cultured for another 2 h at 37°C with 5% CO_2_. The optical density (OD) at 450 nm was measured using a microplate reader.

### Fluorescence *in situ* Hybridization

Online software LncATLAS (http://lncatlas.crg.eu/) was utilized to predict the distribution of GAS5. The subcellular localization of GAS5 was analyzed using a FISH Tag™ RNA green kit with an Alexa Fluortm 488 dye kit (ThermoFisher). The degenerated NPCs were fixed (increasing cell membrane permeability), dehydrated, and hybridized overnight at 42°C. The samples were washed, mixed with 4′,6-diamidino-2-phenylindole, sealed and observed under a confocal microscope (Carl Zeiss, Oberkochen, Germany). Scramble control probe (Scr) was used as a negative control.

### Nuclear-Cytoplasmic RNA Separation

The nuclear RNA and cytoplasmic RNA of cells were extracted using a Cytoplasmic and Nuclear RNA Purification Kit (Amyjet Scientific, Wuhan, China). Cells were dissolved in Lysis Buffer J and centrifuged for 10 min at 4°C at 1,000 × g with the supernatant removed. Cells were then loaded into Spin Column CM and added with a corresponding buffer and ethanol for RNA combination. Subsequently, RNA impurity was washed away with a hypotonic buffer. Then, the Spin Column CM was added with Elution Buffer E to extract target RNA. Finally, the column was incubated for 30 min at 4°C, gently twirled, and centrifuged for 20 min at 15,000 × g. The supernatant obtained was the nuclear RNA.

### Dual-Luciferase Reporter Gene Assay

The Bioinformatics online software Starbase (http://starbase.sysu.edu.cn/index.php) (28) and TargetScan (http://www.targetscan.org/vert_71/) (29) were utilized to predict the binding sites of lncRNA GAS5 with miR-26a-5p, miR-26a-5p, and PTEN. The complementary sequences of lncRNA GAS5 and miR-26a-5p, and miR-26a-5p and PTEN were amplified and cloned into the luciferase reporter plasmid pmiR-GLO luciferase vector (Promega, Madison, WI, USA) to construct wild-type (WT) plasmids (GAS5-WT AND PTEN-WT) and corresponding mutant (MUT) plasmids (GAS5-MUT and PTEN-MUT). The constructed plasmids were cotransfected with mimic NC and miR-26a-5p mimic (GenePharma) into human degenerative NPCs CPH170. After 48 h, the luciferase activity was detected.

### RNA Immunoprecipitation Test

NPCs were lysed with lysis buffer (25 mM Tris-HC1 pH7.4, 150 mM NaC1, 0.5% NP-40, 2 mM EDTA, 1 mM NaF, and 0.5 mM dithiothreitol) containing Rnasin (Takara, Dalian, China) and protease inhibitor mixture (B14001a, Roche, Branchburg, NJ, USA). The lysate was centrifuged for 30 min at 12,000 × g, and the supernatant was isolated and added with anti-Ago2 magnetic beads (130-061-101, Univ-Bio, Shanghai, China), while control group was added with anti-IgG magnetic beads. After incubation for 4 h at 4°C, magnetic beads were washed thrice with washing buffer (50 mM Tris-HC1, 300 mM NaC1 pH7.4, 1 mM MgC1 2, 0.1% NP-40). RNA was extracted from magnetic beads using TRIzol. Expressions of GAS5 and miR-26a-5p were detected by RT-qPCR.

### Western Blot Analysis

The total protein of cells was extracted using a RIPA buffer (Beyotime), and the concentration was determined using a bicinchoninic acid assay kit (Beyotime). The extracted protein was subjected to electrophoresis separation and loaded onto polyvinylidene fluoride membranes. Afterwards, the membranes were blocked with 5% skim milk–Tris-buffered saline-tween (TBST) buffer for 1 h, and incubated overnight with primary antibodies (Table 2) at 4°C. Following three washes with TBST, the membranes underwent a 1-h incubation with secondary antibody goat anti-rabbit IgG H&L (HRP) (1:20,000, ab97051, Abcam) or goat anti-mouse IgG H&L (HRP) (1:5,000, ab205719, Abcam). The membranes were developed using a chemiluminescence reagent, and the ImageJ software (National Institutes of Health, Bethesda, MD, USA) was adopted for gray value analysis of the target bands.

**Table 2:** Primary antibodies used in Western blot.

**Antibody**	**Cat no**.	**Company**	**Dilution**
PTEN	ab267787	Abcam	1:1,000
PI3K (110KD)	ab32089	Abcam	1:1,000
p-PI3K (111KD)	ab125633	Abcam	1:1,000
pan-AKT	ab8805	Abcam	1:500
p-AKT	ab38449	Abcam	1:1,000
Collagen II	ab188570	Abcam	1:1,000
Aggrecan	ab52141	Abcam	1:1,000
MMP-3	ab52915	Abcam	1:1,000
Collagen I	ab138492	Abcam	1:5,000
GAPDH	ab8245	Abcam	1:2,000

### Statistical Analysis

SPSS21.0 (IBM Corp., Armonk, NY, USA) and GraphPad Prism8.0.1 (GraphPad Software Inc., San Diego, CA, USA) were applied for data analysis and figure drawing. All the data conformed to normality distribution and were expressed as mean ± standard deviation. The *t*-test was used for comparison between two groups. One-way analysis of variance (ANOVA) was used for comparison among multiple groups, and Tukey's test was used for *post-hoc* analysis. *p* < 0.05 indicated that the difference was statistically significant.

## Results

### LncRNA GAS5 Is Upregulated in Degenerative NPCs

**Figure 1:**
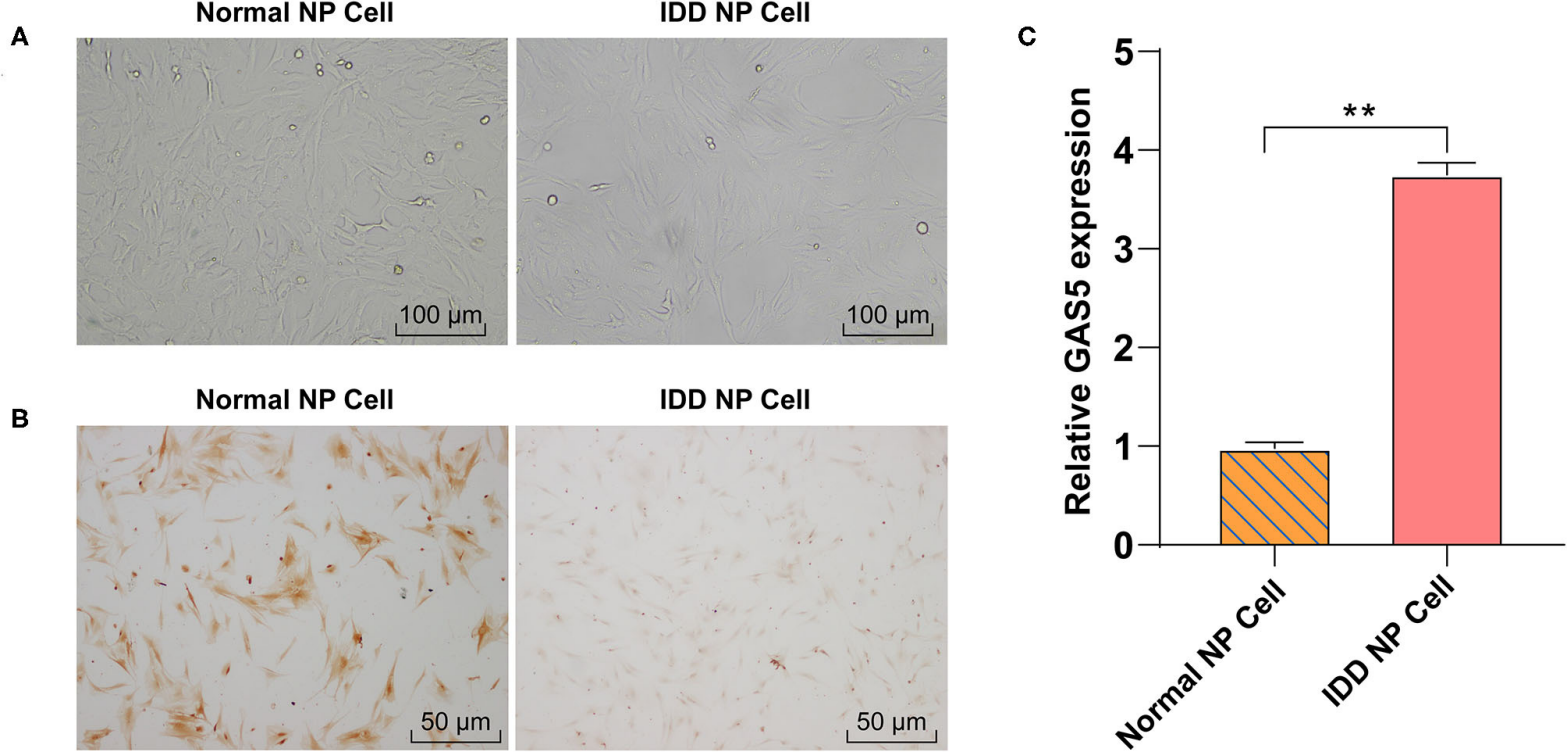
LncRNA GAS5 is upregulated in degenerative NPCs. **(A)** The morphology of degenerative NPCs and normal NPCs was observed under an inverted phase contrast microscope. **(B)** The synthesis of Collagen II (Col II) was detected by immunocytochemistry. **(C)** The expression of GAS5 in degenerative NPCs and normal NPCs was detected by RT-qPCR. The cell experiment was repeated three times, and the data were expressed as mean ± standard deviation; independent *t*-test was used for comparison between groups. **p < 0.01; NP, nucleus pulposus.

It has been reported that GAS5 overexpression may promote the apoptosis of degenerative NPCs (16). However, the synthesis and degradation of ECM of NPC have not been reported. Human degenerative NPCs (CP-H170) and human normal NPCs (CP-H097) were purchased for *in vitro* study. We first observed the morphology of degenerative NPCs and normal NPCs using an inverted phase contrast microscope. Normal NPCs exhibited a clear short fusiform and polygonal morphology while the morphology of degenerative NPCs was an irregular shape of spindle length (Figure 1A). In addition, immunocytochemistry of Col II, the most abundant collagen content in NP tissues, showed that the cytoplasm of normal NPC was dyed dark brownish yellow while the cytoplasm of degenerative NPC was dyed light yellow, which was consistent with the decrease in Col II synthesis function (Figure 1B). These results verified the correctness and availability of NPCs. We then detected GAS5 expression in NPCs by RT-qPCR. GAS5 expression in degenerative NPCs was higher than that in normal NPCs (Figure 1C, *p* < 0.01). These results suggest that the up-regulation of GAS5 expression may be related to IDD occurrence.

### Inhibition of GAS5 Enhances the Viability of Degenerative NPCs and Promotes ECM Synthesis

**Figure 2:**
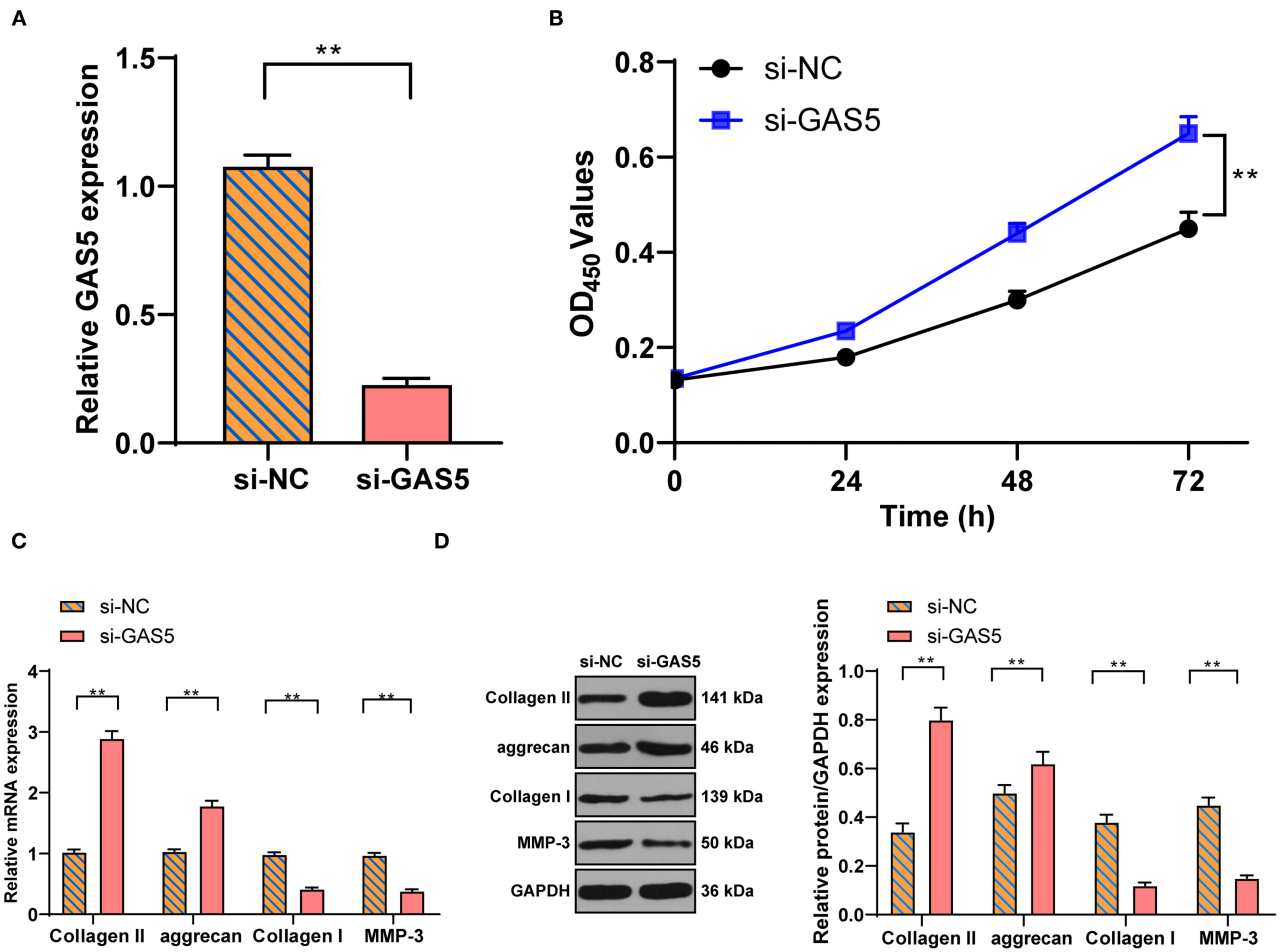
Inhibition of GAS5 enhances the viability of degenerative NPCs and promotes ECM synthesis. Si-GAS5 was transfected into degenerative NPCs, the transfected si-NC was used as the control, and then the expression of GAS5 in degenerative NPCs was detected by **(A)** RT-PCR to verify the transfection efficiency; then **(B)** CCK-8 test was used to detect the survival rate of degenerative NPCs; **(C,D)** RT-PCR and Western blot were used to detect the expression of ECM-related genes in NP. The cell experiment was repeated three times, and the data were expressed as mean ± standard deviation and analyzed using the independent *t*-test; **p < 0.01.

To study the action of GAS5 in degenerative NPCs and ECM, si-GAS5 was transfected into degenerative NPCs to inhibit GAS5 expression. RT-qPCR exhibited that GAS5 expression in degenerative NPCs was effectively reduced after si-GAS5 transfection (Figure 2A, *p* < 0.01). CCK-8 analysis showed that compared with the si-NC group, the proliferation of degenerative NPCs in the si-GAS5 group was significantly enhanced (Figure 2B, *p* < 0.01). The mRNA and protein levels of ECM-related genes were further detected by RT-qPCR and Western blot. Compared with the si-NC group, the levels of Collagen II and aggrecan were upregulated, while the levels of Collagen I and MMP-3 were downregulated in the si-GAS5 group (Figures 2C,D, all *p* < 0.01). In brief, downregulation of GAS5 expression can improve the proliferation of degenerative NPCs, increase ECM synthesis, and inhibit the ECM degradation of degenerative NPCs.

### LncRNA GAS5 and PTEN Competitively Bind to miR-26a-5p

**Figure 3:**
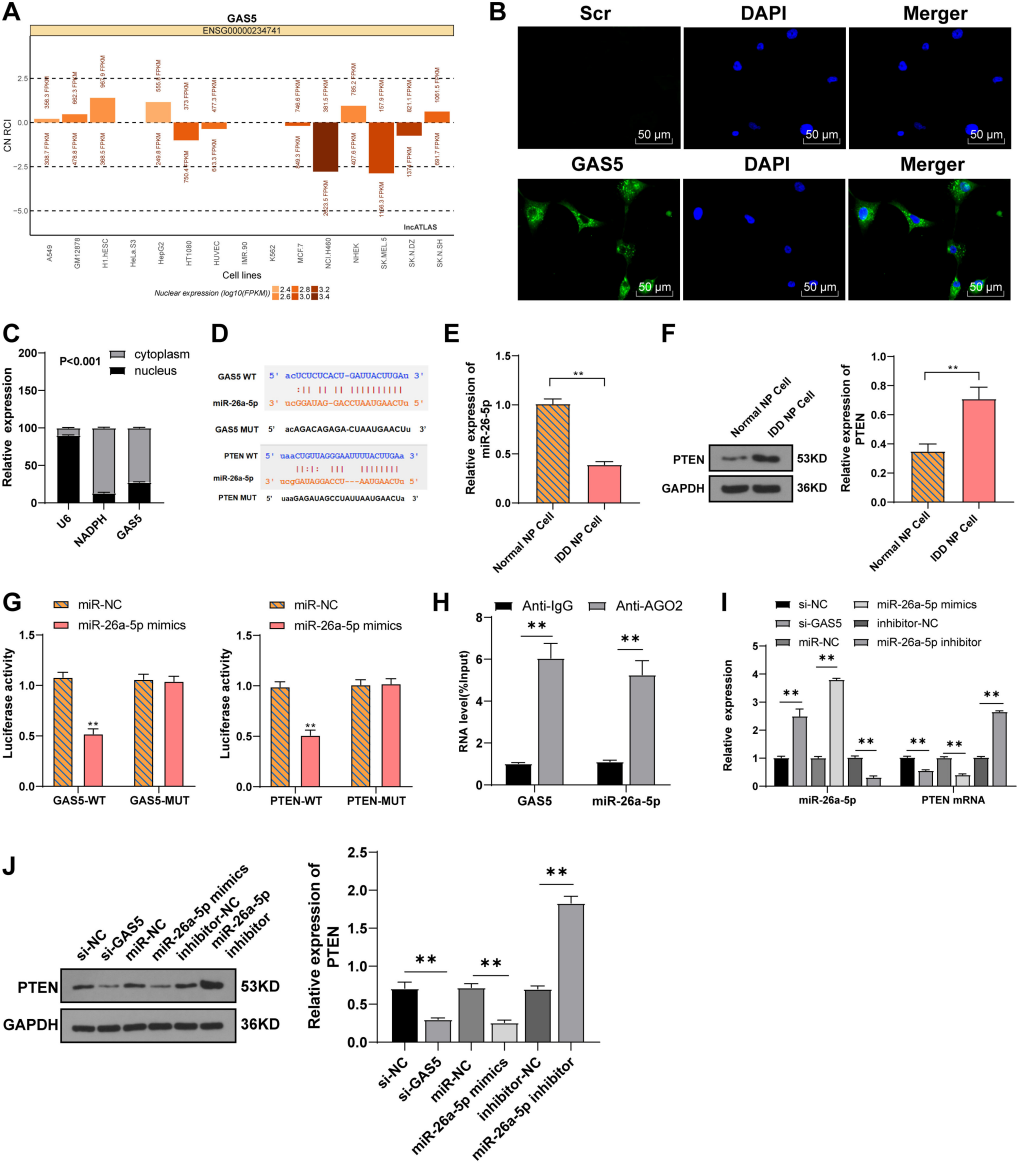
LncRNA GAS5 and PTEN competitively bind to miR-26a-5p. **(A)** LncATLAS predicted that lncRNA GAS5 was mostly located in the cytoplasm. **(B)** FISH assay and **(C)** nuclear-cytoplasmic RNA separation confirmed the distribution of lncRNA GAS5 in degenerative NPCs. **(D)** Starbase and TargetScan predicted the binding sites between lncRNA GAS5 and miR-26a-5p, and miR-26a-5p and PTEN. **(E)** RT-qPCR was used to detect miR-26a-5p expression. **(F)** Western blot was used to detect PTEN expression. **(G)** Dual-luciferase assay confirmed the binding relation between lncRNA GAS5 and miR-26a-5p, and miR-26a-5p, and PTEN. **(H)** RIP test verified the interaction between lncRNA GAS5 and miR-26a-5p. **(I)** RT-qPCR was used to detect the miR-26a-5p expression and PTEN mRNA expression in degenerative NPCs. **(J)** Western blot was used to detect PTEN expression in degenerative NPCs. The cell experiment was repeated three times, and the data were expressed as mean ± standard deviation; independent *t*-test was used for comparison between two groups in **(E–H)**, while one-way ANOVA was used for comparison among multiple groups in **(I,J)**, followed by Tukey's multiple comparisons test. **p < 0.01.

The action mechanism of lncRNA relies on its subcellular localization. In order to study the mechanism of GAS5 in degenerative NPCs, we first predicted with the LncATLAS software that GAS5 might mainly be located in the cytoplasm (Figure 3A), while the FISH experiment and nuclear-cytoplasmic RNA separation further clarified that GAS5 was mainly in the cytoplasm of NPCs (Figures 3B,C), suggesting that lncRNA GAS5 may act as a ceRNA in IDD. The online software Starbase was employed to predict the ceRNA network of GAS5. It was predicted that there were binding sites between GAS5 and miR-26a-5p, and between miR-26a-5p and PTEN (Figure 3D). As reported, PTEN can promote IDD by regulating the behaviors of degenerative NPCs (23). miR-26a-5p was reported to be the target of PTEN in myocardial injury (26). RT-qPCR and Western blot showed that miR-26a-5p expression was decreased in degenerative NPCs (Figure 3E, *p* < 0.01), while PTEN expression was increased in degenerative NPCs (Figure 3F, *p* < 0.01). Therefore, we speculated that GAS5 may play a role in degenerative NPCs by binding to miR-26a-5p to regulate PTEN. Dual-luciferase assay confirmed the binding relationship between GAS5 and miR-26a-5p, and miR-26a-5p and PTEN (Figure 3G, *p* < 0.01). RNA-induced silencing complex (RISC) is a ribonucleoprotein complex containing argonaute (AGO) and other proteins that bind to mature miRNAs and post-transcriptionally mediate gene silencing or the degradation of mRNA target genes, and thus participate in gene expression regulation (30). Therefore, we performed the RIP test using AGO2 antibody magnetic beads, and the result showed enrichments of lncRNA GAS5 and miR-26a-5p in AGO2 RIPs (Figure 3H, *p* < 0.01), suggesting that the pull-down of AGO2 protein and the miR-26a-5p binding to it can lead to the pull-down of GAS5 binding to RISC. Therefore, we speculated that GAS5 and miR-26a-5p were in the same AGO2 complex in NPCs. Dual-luciferase reporter assay showed that GAS5 and PTEN competitively bound to miR-26a-5p. Then, the expression of miR-26a-5p and PTEN after GAS5 knockdown was detected by RT-qPCR and Western blot. Compared with the si-NC group, miR-26a-5p expression was upregulated, while mRNA and protein expressions of PTEN were downregulated in the si-GAS5 group (Figures 3I,J, all *p* < 0.01). Then, the miR-26a-5p mimic/inhibitor was transfected into degenerative NPCs. miR-26a-5p and PTEN expressions were detected. Compared with the miR-NC group, miR-26a-5p expression was upregulated and PTEN mRNA expression was downregulated in the miR-26a-5p mimic group, while the expression trend was opposite to that in the miR-26a-5p inhibitor group (Figures 3I,J, all *p* < 0.01). Taken together, GAS5 in degenerative NPCs can act as a ceRNA to compete with PTEN to bind to miR-26a-5p.

### Overexpression of miR-26a-5p Promotes ECM Synthesis in Degenerative NPCs

**Figure 4:**
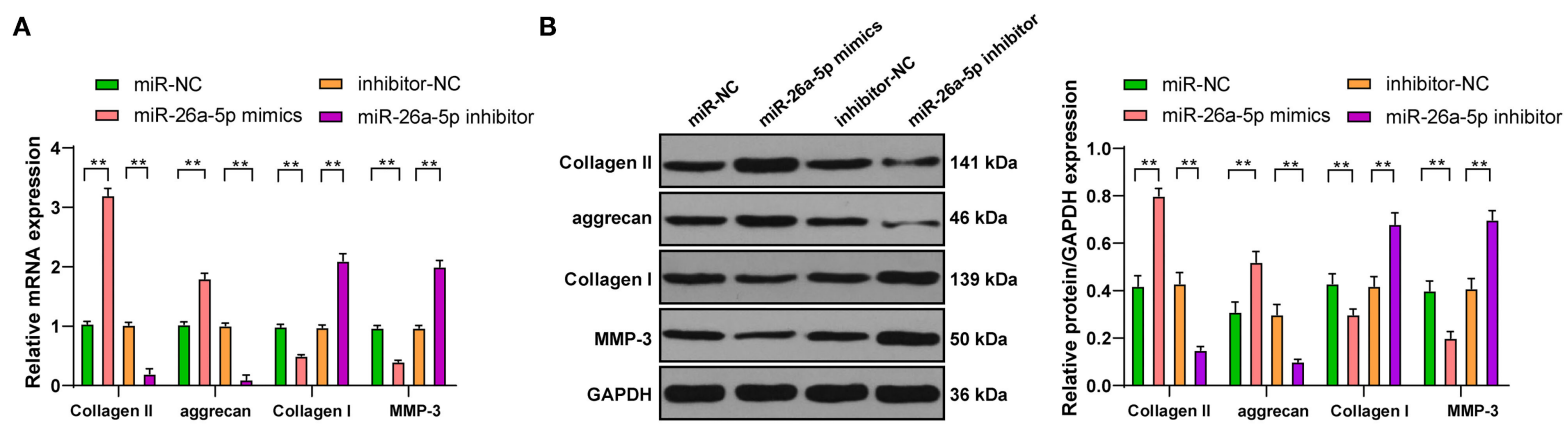
Overexpression of miR-26a-5p can promote ECM synthesis in degenerative NPCs. In degenerative NPCs transfected with miR-26a-5p mimic/inhibitor, the mRNA, and protein levels of ECM-related genes were detected by **(A)** RT-qPCR and **(B)** Western blot. The cell experiment was repeated three times, and the data were expressed as mean ± standard deviation and analyzed using one-way, followed by Tukey's multiple comparisons test. **p < 0.01.

To investigate the action of miR-26a-5p on the ECM of NPCs, RT-qPCR, and Western blot were used to detect the levels of genes related to the ECM of degenerative NPCs that overexpressed or interfered with miR-26a-5p. Compared with miR-NC group, the levels of Collagen II and aggrecan were clearly upregulated, and the levels of Collagen I and MMP-3 were significantly downregulated in degenerative NPCs with miR-26a-5p overexpression, while the levels of ECM-related genes in miR-26a-5p inhibitor group were opposite (Figures 4A,B, all *p* < 0.01). In conclusion, overexpression of miR-26a-5p can intensify ECM synthesis in degenerative NPCs.

### LncRNA GAS5/miR-26a-5p/PTEN Regulates ECM Synthesis of Degenerative NPCs via the PI3K/Akt Pathway

**Figure 5:**
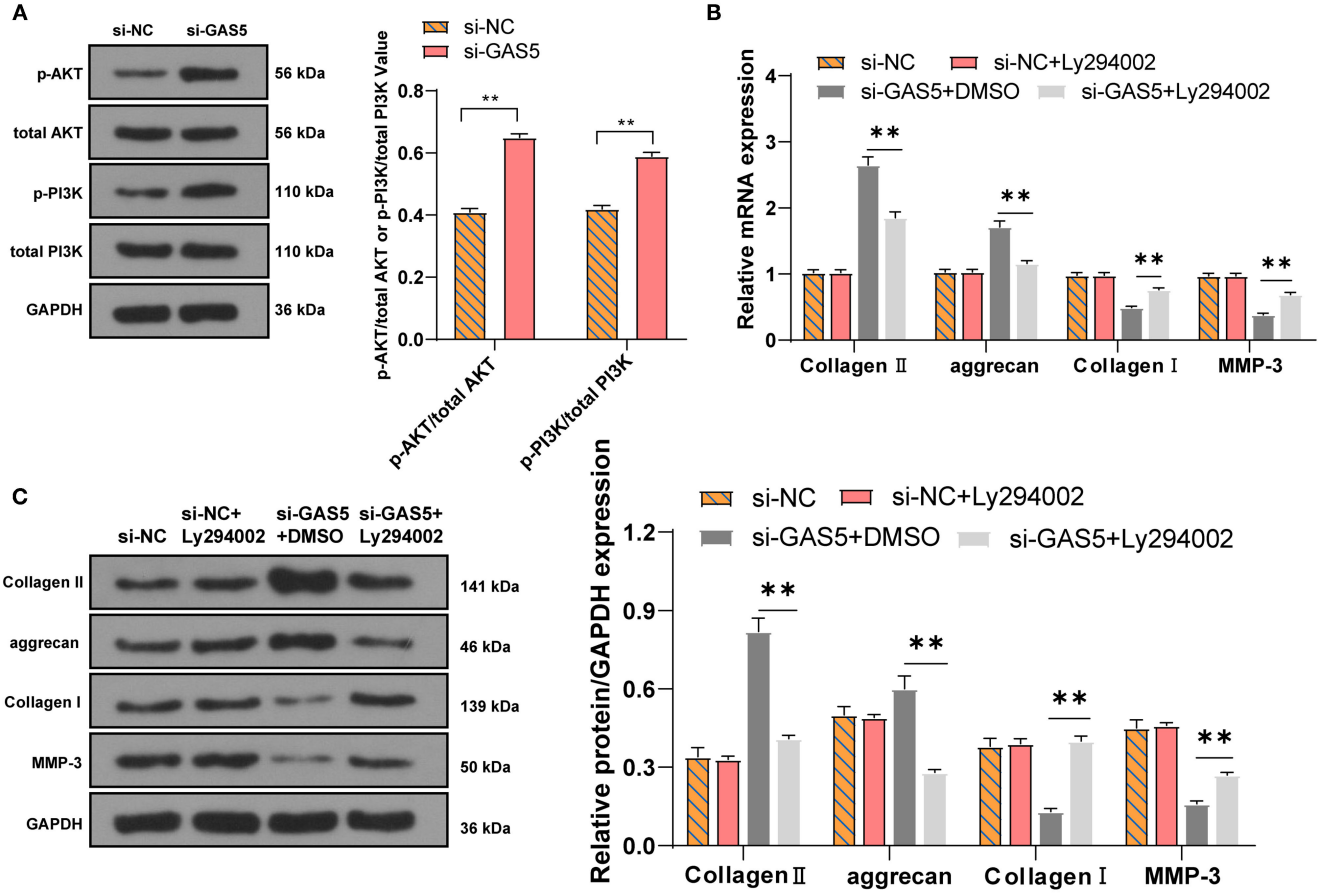
LncRNA GAS5/miR-26a-5p/PTEN regulates the ECM synthesis of degenerative NPCs *via* the PI3K/Akt pathway. **(A)** The protein levels of PI3K/Akt in degenerative NPCs with low expression of GAS5 were detected by Western blot; the PI3K/Akt inhibitor LY294002 was added to degenerative NPCs with low expression of GAS5, and si-GAS5 + DMSO was used as control. **(B,C)** RT-qPCR and Western blot were used to detect the level of ECM-related genes. The cell experiment was repeated three times, and the data were expressed as mean ± standard deviation; independent *t*-test was used for comparison between two groups in **(A)** while one-way ANOVA was used for comparison among multiple groups in **(B,C)**, followed by Tukey's multiple comparisons test. **p < 0.01.

We further explore the downstream pathway. PTEN has a classical negative regulatory relationship with the PI3K/Akt pathway (31). We then detected the activation of PI3K/Akt pathway in degenerative NPCs with GAS5 knockdown by Western blot. Compared with si-NC group, the levels of p-PI3K/total PI3K and p-Akt/total Akt in the si-GAS5 group were significantly increased (Figure 5A, *p* < 0.01). Then we set up a rescue experiment: LY294002, a PI3K/Akt inhibitor, was added to the degenerative NPCs with GAS5 knockdown. The levels of ECM-related genes in NPCs were detected. Compared with the si-GAS5 + DMSO group, the levels of Collagen II and aggrecan in the si-GAS5 + LY294002 group were decreased, while the levels of Collagen I and MMP-3 were clearly elevated (Figures 5B,C, all *p* < 0.01). In short, inhibition of the Akt pathway may inhibit the ECM synthesis of degenerative NPCs promoted by GAS5 knockdown. Collectively, the GAS5/miR-26a-5p/PTEN axis could regulate the synthesis and degradation of ECM in degenerative NPCs *via* the PTEN/Akt pathway.

## Discussion

In recent years, with the aging of the population, the incidence rate of spinal degenerative diseases has increased rapidly; currently, the treatment is mainly focused on the relief of clinical symptoms rather than the recovery of underlying pathophysiological processes (16). Therefore, further investigation of IDD is urgently needed. This study highlighted that lncRNA GAS5 was overexpressed in IDD, and GAS5 can compete with PTEN to bind to miR-26a-5p, thus inhibiting ECM the synthesis of degenerative NPCs.

A number of lncRNAs are deregulated in NPCs, suggesting the importance of lncRNAs in IDD development (32, 33). But the expression pattern of GAS5 in NPCs is rarely studied. Our data revealed that GAS5 expression in degenerative NPCs was higher than that in normal NPCs. A previous microarray study has demonstrated GAS5 overexpression in degenerative NPCs (33). These results suggested that up-regulation of GAS5 may be related to IDD occurrence. The vast majority of cartilage volume is ECM, which is predominantly composed of Collagen II and aggrecan (34). The increasing grade of degeneration facilitates the expression of MMPs, which further degrades Collagen II and aggrecan and aggravates spinal instability (35). At the beginning of IDD, the IVD transfers to a catabolic mode, with reduced expression of aggrecan and Collagen II and increased activity of MMP-3 (36–38). Importantly, GAS5 is associated with modulation of ECM anabolism and catabolism in chondrocytes (13). The ectopic expression of GAS5 in human osteoarthritis chondrocytes elevates MMP-3 expression and reduces the contents of Collagen II and aggrecan (39). To study the role of GAS5 in the changes of ECM, si-GAS5 was transfected into degenerative NPCs. After GAS5 knockdown, the proliferation of degenerative NPCs was significantly enhanced, the levels of Collagen II and aggrecan were upregulated, while the levels of Collagen I and MMP-3 were downregulated. Downregulation of MMP-3 indicates the disorder of ECM composition and a more balanced anabolism/catabolism (40, 41). Aggrecan is the most abundant proteoglycan secreted by NPCs, which in turn enables the compressive capacity of NPCs (42). The obviously promoted degenerative NPC proliferation was accompanied by increased expression of aggrecan and Collagen II (40). Consistently, overexpression of GAS5 elevates MMP-3 expression and stimulates the apoptosis of chondrocytes (39). Knockdown GAS5 augments ECM accumulation, associated with collagen deposition (43, 44). In brief, downregulation of GAS5 can improve the proliferation and ECM synthesis of degenerative NPCs.

We then aimed to figure out the molecular mechanism of GAS5 in IDD. The LncATLAS software and FISH experiment confirmed that GAS5 was mainly located in the cytoplasm of NPCs, suggesting the regulatory network of ceRNA of GAS5 in IDD. We further found binding sites between GAS5 and miR-26a-5p, and between miR-26a-5p and PTEN. miR-26a is notably downregulated in human degenerative NPCs (45). PTEN can promote IDD by regulating the behaviors of degenerative NPCs (23). Dual-luciferase assay confirmed the binding relationship between GAS5 and miR-26a-5p, and miR-26a-5p and PTEN. GAS5 is also identified as a miR-26b-5p sponge to increase PTEN expression in human aortic vascular smooth muscle cells (20). In conclusion, GAS5 in degenerative NPCs can act as a ceRNA to compete with PTEN to bind to miR-26a-5p. Furthermore, miR-26a is closely related to the differentiation and development of bones and it modulates ECM homeostasis in cartilage (46). To investigate the action of miR-26a-5p on ECM of NPCs, we detected the levels of genes related to the ECM of degenerative NPCs that overexpressed or interfered with miR-26a-5p. The levels of Collagen II and aggrecan were clearly upregulated, while the levels of Collagen I and MMP-3 were significantly downregulated in degenerative NPCs with miR-26a-5p overexpression. miR-26a promotes the expression of Collagen II and aggrecan in chondrocytes, thus repressing ECM degradation (47). In summary, overexpression of miR-26a-5p can intensify ECM synthesis in degenerative NPCs. We further explore the downstream pathway. PTEN is overexpressed in degenerative NPCs, and PTEN inhibits the production of ECM components including Collagen II, aggrecan, and proteoglycan Akt (23). PTEN has a classical negative regulatory relationship with the PI3K/Akt pathway (31). The phosphorylation levels of PI3K and Akt in degenerative NPCs with GAS5 knockdown were significantly increased. Then we set up a rescue experiment: LY294002, a PI3K/Akt inhibitor, was added to the degenerative NPCs with GAS5 knockdown. The levels of Collagen II and aggrecan in the si-GAS5 + LY294002 group were significantly decreased, while the levels of Collagen I and MMP-3 were clearly elevated. Inhibition of PTEN and activation of Akt are beneficial to prevent IDD degradation and prevent NPC apoptosis (48). miR-26a-5p also targets PTEN to activate the PI3K/AKT pathway in cardiomyocytes (26). In short, inhibition of Akt pathway may inhibit the ECM synthesis of degenerative NPCs promoted by GAS5 knockdown.

Collectively, our study may provide a possible mechanism for GAS5 as a crucial regulator in IDD. We initially demonstrated that the GAS5/miR-26a-5p/PTEN axis could regulate the synthesis and degradation of ECM in degenerative NPCs *via* the PTEN/Akt pathway. GAS5-based medical interventions may be effective approaches for the therapy of IDD. Although the present study provided therapeutic value for IDD management, the experiment results and clinical application need to be further verified. Certain limitations exist in this study. First, although the correctness and availability of NPCs we purchased were verified by observing cell morphology and detecting Col II synthesis, detailed clinical information of NPCs were not included. Isolating NPCs from clinical IDD tissue samples for *in vitro* study would be more accurate. Recently, genetic mapping assays have been used to generate NPC from human pluripotent stem cells as valuable NPC sources (49). Second, the definition of degenerative NPCs is lacking; similar to aging MSCs, degenerative NPCs should present some common features of senescence, i.e., mitochondrial dysfunction or genomic aging markers (50). In addition, inappropriate activation of the NFκB signaling pathway is commonly observed in aging NPCs and MSCs that will affect normal paracrine function including lncRNAs and ECM secretion (51, 52). Moreover, this study only included *in vitro* experiment, and *in vivo* experiment was not conducted. In future studies, we will further verify the *in vivo* functional mechanism of lncRNA GAS5 based on the animal model available for the study of IDD (53).

## Data Availability Statement

The raw data supporting the conclusions of this article will be made available by the authors, without undue reservation.

## Author Contributions

LT: conceptualization. YX and YY: validation, data reviewing, and writing. KH: review and editing. All authors read and approved the final manuscript.

## Conflict of Interest

The authors declare that the research was conducted in the absence of any commercial or financial relationships that could be construed as a potential conflict of interest.

## Publisher's Note

All claims expressed in this article are solely those of the authors and do not necessarily represent those of their affiliated organizations, or those of the publisher, the editors and the reviewers. Any product that may be evaluated in this article, or claim that may be made by its manufacturer, is not guaranteed or endorsed by the publisher.
